# A signal transduction blind spot: the function of adenylyl cyclase transmembrane domains

**DOI:** 10.1111/febs.70022

**Published:** 2025-02-12

**Authors:** Ryan S. Dowsell, Matthew G. Gold

**Affiliations:** ^1^ Department of Neuroscience, Physiology & Pharmacology University College London UK

**Keywords:** adenylyl cyclase, cAMP, protein–protein interaction, signal transduction, transmembrane domain

## Abstract

Signal transduction of external primary signals into intracellular elevations of the second messenger cyclic AMP is an ancient and universal regulatory mechanism in biology. In mammals, 9 of the 10 adenylyl cyclases (ACs) share a common topology that includes a large transmembrane (TM) domain assembled from two clusters of six helices. This domain accounts for ~ 35% of the coding sequence but, remarkably, its function is still an open question. In this viewpoint, we consider how the first primary AC sequences spurred ideas for the purpose of AC TM domains, including voltage‐sensing and transporter functions. In the original conceptions of second messenger signalling, ACs were put forward as potential receptors, and we discuss emerging evidence in support of this function. We also consider growing evidence that cyclase TM helical bundles help to organise multiprotein signalling complexes by engaging in interactions with other membrane‐embedded proteins. Cyclase TM regions are more diverse between isoforms than the catalytic domain—we conclude by considering how this might be exploited in therapeutic strategies targeting specific cyclase isoforms.

## Graphical abstract

Transmembrane adenylyl cyclases (tmACs) are essential for synthesising the universal second messenger cyclic AMP. All tmACs share a common topology that includes a large 12‐helix transmembrane (TM) domain. This domain accounts for ~ 35% of the coding sequence but its function is still an open question. In this viewpoint, we consider whether cyclase TM domains might function as transporters, voltage sensors, receptors and interaction modules.

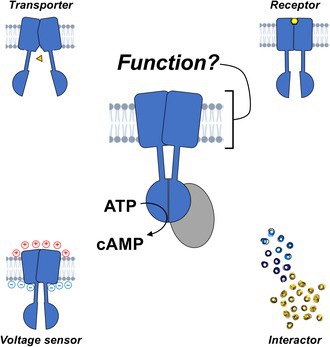

**Abbreviations**
ABC: ATP‐binding cassette
AC: adenylyl cyclase
AKAP: A‐kinase anchoring protein
CAI‐1: cholerae autoinducer‐1
cAMP: cyclic 3′‐5′ adenosine monophosphate
CB1: cannabinoid receptor 1
CREB: cAMP response element‐binding protein
GPCR: G protein‐coupled receptor
GWAS: genome‐wide association study
NBD: nucleotide‐binding domain
PDE: phosphodiesterase
PKA: protein kinase A
PPAR: peroxisome proliferator‐activated receptors
SDPA: 1‐stearoyl‐2‐docosahexaenoyl‐phosphatidic acid
TM: transmembrane
tmAC: transmembrane adenylyl cyclase

## Introduction

**Fig. 1:**
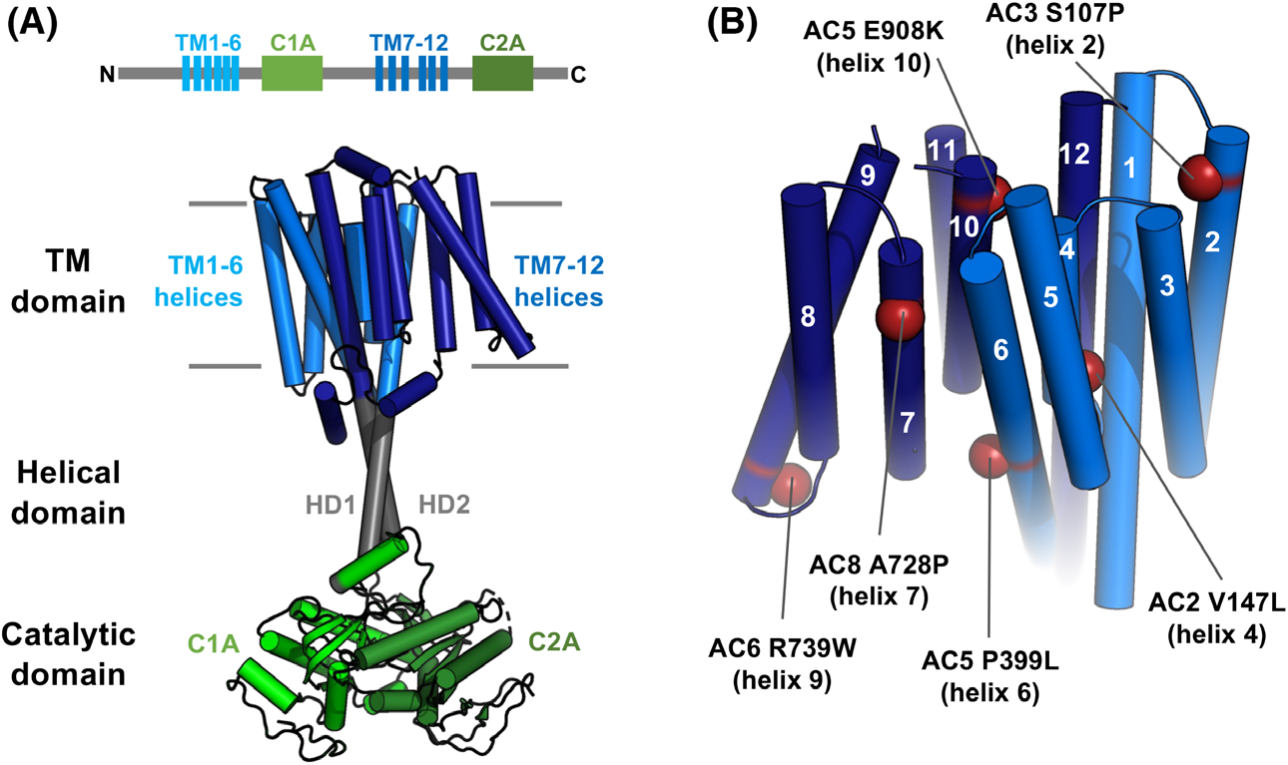
Transmembrane adenylyl cyclase (tmAC) topology and locations of transmembrane (TM) domain pathogenic mutations. (A) Mammalian tmAC structure showing TM (blue), helical (grey) and catalytic (green) domains. The structure corresponds to AC8 (PDB 8BUZ). (B) Pathogenic mutations falling within the TM domain are shown mapped onto the AC8 structure (PDB 8BUZ) with reference to the AC5 cryo‐EM structure (PDB 8SL3), and AlphaFold models for AC2, 3 and 6. The mutations have the following disease associations: AC2 V147L, bipolar disorder [12]; AC3 S107P, childhood obesity [13]; AC5 P399L, early‐onset generalised dystonia [14]; AC5 E908K, spastic paraparesis [15]; AC6 R739W, autism [16]; and AC8 A728P, schizophrenia [17]. Structural representations were generated using pymol (Schrodinger Inc, New York, NY, USA).

Cyclic adenosine 3′,5′‐monophosphate (cAMP) is a universal second messenger that mediates intracellular responses including changes in synaptic strength, cell contractility, permeability, migration and differentiation [1]. In the first conception of a second messenger in the 1960s, membrane‐embedded adenylyl cyclase (AC) was ear‐marked as a potential receptor for hormones (primary messengers), with the cyclase directly transducing these signals into the production of the intracellular second messenger cAMP [2]. Gradually a canonical pathway has developed with extracellular signalling molecules acting on G protein‐coupled receptors (GPCRs) that activate transmembrane ACs (tmACs) via G proteins [3]. There are nine tmACs in mammals—all nine share a similar structure (Fig. 1A) [4, 5]. The cyclases are comprised of two major regions—catalytic (green) and transmembrane (TM, blue) regions that are linked by ~ 40‐amino acid extended helices (grey). Both catalytic and TM regions are assembled from two halves that are separated in the primary sequence [6, 7]: subdomains C1A and C2A form a pseudo‐dimer that constitutes the catalytic domain (green, Fig. 1A) whereas two clusters of 6 helices assemble into a contiguous 12‐helix TM domain (blue) [8, 9, 10]. The first report of an adenylyl cyclase primary sequence in 1989 triggered a wave of studies focusing on possible functions for the TM regions beyond membrane tethering [11]. These studies were spurred by sequence similarities to ATP‐binding cassette (ABC) transporters [11] and from the intuition that evolution would be unlikely to maintain ~ 35% of the coding sequence to a domain that served no purpose beyond what could be achieved with a short signal sequence. The importance of these domains is underscored by several pathological mutations located in AC TM domains (Fig. 1B) [12, 13, 14, 15, 16, 17]. However, with no clear function emerging, research has focused on more tractable aspects of AC regulation involving intracellular elements. For example, the catalytic mechanism is well understood [4], as are regulation of the catalytic region by direct binding to G proteins [6] and the basis of interactions between intracellular cyclase elements and A‐kinase anchoring proteins (AKAPs) that organise cAMP signalling in subcellular compartments [18].

The last 5 years have seen the first high‐resolution structures of full‐length tmACs [8, 9, 10, 19]. The structures confirm that the TM domain is physically separated from the catalytic region and comprised of two integrated six‐helix bundles, with each bundle connected to half of the catalytic domain via half of a coiled‐coil helical domain (Fig. 1A). The structures have the potential to serve as a catalyst for finding answers to the old problem of assigning a function to the mammalian cyclase TM domain. They provide more information to assess the likelihood of different theories and simply serve as a potent visual reminder that the problem still exists. The aim of this viewpoint article is to introduce different possible functions put forward for cyclase TM domains before considering two functions in detail and how this research area could support isoform‐specific AC pharmacology.

## Origin of putative functions for the TM domain

**Fig. 2:**
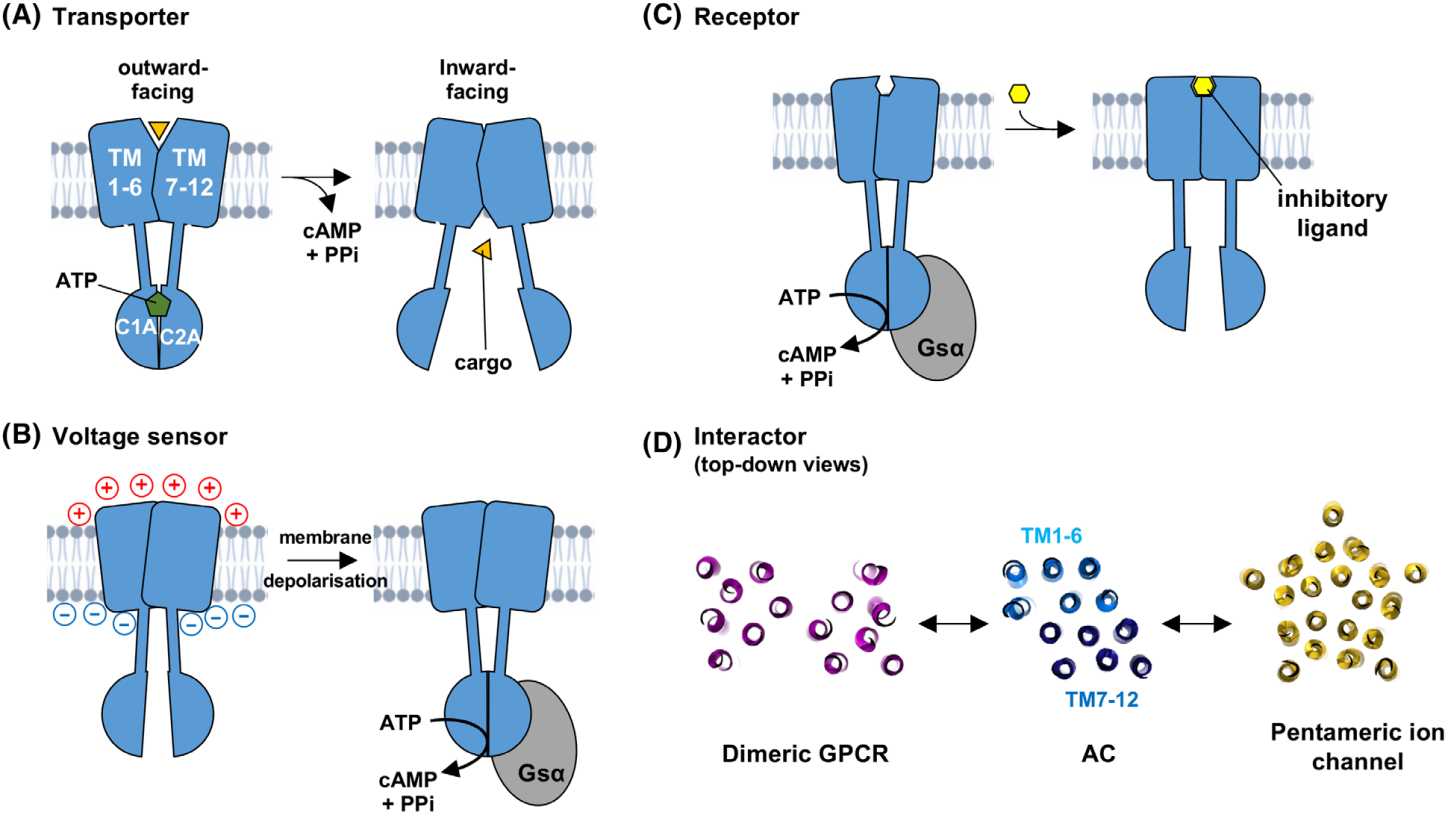
Putative functions of the adenylyl cyclase (AC) transmembrane (TM) domain. (A) ATP hydrolysis could be coupled to a change from an outward to an inward‐facing pose to support transport of a molecule across the membrane. (B) Changes in membrane polarisation could be sensed by the TM domain to alter the sensitivity of the catalytic regions to, for example, regulatory G proteins. (C) The TM domains may serve a receptor function, for example, enabling allosteric inhibition as shown. (D) The 12 helices of the TM domain are well‐placed to engage in interactions with helices present in other integral membrane proteins including GPCRs and ion channels.

The first mammalian tmAC primary sequence revealed a striking topological similarity to the transporter P‐glycoprotein [11]. P‐glycoprotein is a prototypic member of the ABC transporter family, now known to contain 48 members in humans [20], and all transporters in this family share the same tmAC‐like topology. The ATP hydrolysis site in the transporters is located between two catalytic subdomains—identical to the structural arrangement of the tmAC catalytic interface for ATP cyclisation. ABC transporters also contain polytopic TM domains comprised of two integrated six‐helix bundles. ATP hydrolysis drives the transition of the ABC transporter TM domain between inward and outward‐facing conformations, triggering the translocation of substrates across the plasma membrane [21]. This similarity hints at a transporter function in mammalian tmACs (Fig. 2A)—if so, what might be the cargo? Interestingly, the *Dictyostelium discoideum* (*D. discoideum*) ABC transporter, abcB3, is one of several transporters known to export cAMP as a chemotactic signal for aggregation [22]. There are some emerging functions for extracellular cAMP in mammals, however, four ABC transporters including MRP4 are already thought to export cAMP [23]. Structures of MRP4 have been determined at different stages of the export cycle [24], focusing on the transporter's ability to export prostanoids. In the apo state with neither ATP nor substrate bound, the two pseudo‐dimeric halves of the transporter flex open. In this state, the two nucleotide‐binding domains (NBDs) are separated by ~ 25 Å, and a deep wedge‐shaped cavity is revealed between the two TM subdomains. The substrate binds at the base of this cavity, bringing about partial closure of the clamshell‐like structure—closure is completed upon binding of ATP to the NBDs [24]. It is notable that no substrate exit channel is evident in the closed ATP‐bound MRP4 structure [24]. To date, all tmAC structures resemble the ‘closed’ MRP4 conformation, with the catalytic subdomains bound to one another and the two TM subdomains oriented in parallel. We await tmAC structures in the absence of activators that might reveal whether the cyclases can open to reveal cavities on the internal face of the TM domain that could support transport. After 35 years, there are still no reports of any transporter activity for a mammalian tmAC, and it should be noted that the topographical similarity to ABC transporters is not complemented by substantial primary sequence similarity.

Krupinski et al. [11] also immediately noted a similarity to ‘dihydropyridine‐sensitive Ca^2+^ channels and K^+^ channels’. The possibility of an ion channel or voltage‐sensor function was reinforced by the report of a *Paramecium tetraurelia* AC that responds to membrane hyperpolarisation [25]. Remarkably, the Paramecium cyclase is not only regulated by the membrane potential but is itself the ion channel responsible for setting the resting potential [25]. However, it appears that these dual‐function cyclases are restricted to protozoans [25, 26]. The TM domains of ion channels typically contain channels lined by charged amino acids that enable ion flux—such features are absent in mammalian tmACs. Could the TM domains at least enable cyclase regulation via changes in membrane potential (Fig. 2B)? Experiments in primary neurons from rat cerebellum and hippocampus showed that KCl‐mediated membrane depolarisation can cooperate with isoproterenol to elevate cAMP [27], suggestive of the presence of a voltage‐sensing cyclase. However, it was subsequently shown that this effect resulted from Na^+^ entry through L‐type Ca^2+^ channels [28]. Voltage‐gated ion channels are typically activated by positively charged ‘S4’ TM helices that move in response to changes in membrane potential [29]. Although such sequences are absent in tmACs, a voltage‐sensing function cannot be ruled out since the ligand‐sensitivity of several GPCRs is regulated by membrane depolarisation in the absence of S4‐type helices [30].

The following sections focus on functions where the balance of evidence indicates that TM domains can act in this way to at least some extent: as receptors for ligands whose binding is coupled to changes in cyclase activity (Fig. 2C); and as modules for interaction with other integral membrane proteins (Fig. 2D).

## Receptor function

How plausible is it that binding of a ligand to the TM region could be coupled to either inhibition (Fig. 2C) or activation of cyclase activity? Experiments with chimeric and split tmACs demonstrate that both halves of the TM domain must be properly aligned to support cyclase activity. Isolated C1 and C2 subdomains from AC8 co‐expressed in HEK293 cells are not able to combine to support catalysis whereas co‐expression of AC8 M1‐C1 and M2‐C2 fragments generates an active cyclase [31]. Reinforcing the idea that the two TM subdomains are able to dictate whether the catalytic domains are positioned in a conformation compatible with catalysis, AC5/AC6 chimeras containing swapped TM subdomains exhibit normal localisation and expression levels but are effectively catalytically dead [32]. The helical domain appears well‐placed to conduct structural changes between the TM and catalytic regions. Interestingly, several pathogenic mutations have been identified within the helical domain [7] including gain‐of‐function mutations at residue R418 in AC5 that have been linked to familial dyskinesia [8]. Studies of helical domain mutations in *Mycobacterium intracellulare* AC Cya [33], and *D. discoideum* AC [34] reinforce the idea that changes in the conformation of the helical domains can control cyclase activity in both directions. The equivalent domain is also critical for catalysis in guanylate cyclases [35]. Proof‐of‐concept studies with synthetic cyclases sensitive to TM domain ligands reinforce the idea that a receptor function for the TM domains is possible. Swapping the TM domain of the *Mycobacterium tuberculosis* AC Rv1625c for the equivalent region of the *Vibrio harveyi* quorum‐sensing histidine kinase CqsS generates a chimeric cyclase that can be directly stimulated by the lipophilic ligand cholerae autoinducer‐1 (CAI‐1) [36]. A similar strategy has also been applied to AC2. In this case, exchanging the TM domains with the equivalent CqsS sequence generates a cyclase that is inhibited by CAI‐1 [37]. Taken together, these studies indicate that structural changes elicited by a TM domain ligand could conceivably be transmitted to the catalytic domain in mammalian tmACs to either increase or decrease cyclase activity.

A limited number of molecules have been put forward as allosteric modulators that act through the TM domain. Miconazole, and the related fungicidal imidazole‐containing compounds econazole and clotrimazole, have been shown to alter activity in HEK293 membrane extracts, with a proposed site of action outside of the catalytic domain [38]. Whereas miconazole inhibits AC1 and AC2 and activates AC9, clotrimazole inhibits AC1 and AC9, and econazole stimulates AC9 [38]. It should be noted that there is no conclusive evidence that these compounds act via the TM domain, and the related imidazole compound calmidazolium inhibits a cyclase construct limited to the catalytic region [39]. In recent years, the Schultz laboratory has identified several lipids that alter AC activity. Lipids are obvious candidates for TM domain modulation given their ability to regulate many other integral membrane proteins [40]. Building on the observation that G_s_α stimulation of several tmACs is inhibited by serum [37], both AC inhibitors and activators have been identified in lipid extracts from serum. The fatty acid 1‐stearoyl‐2‐docosahexaenoyl‐phosphatidic acid (SDPA) was found to potentiate G_s_α‐stimulated AC3 activity [41] but it did not affect an isolated catalytic domain pairing (AC1 C1 subdomain with AC2 C2 subdomain). The authors proposed an intracellular TM domain binding site in this study [41]. Heme b was identified in a low‐pH extract from the lung as an inhibitor of multiple cyclase isoforms [42] and hemin (heme b chloride) inhibited multiple AC isoforms in HEK293 cells. Hemin inhibition of membranes containing cyclase was found to be reversible, suggesting that heme exerts its effects by binding directly to ACs rather than by remodelling of the membrane. Most recently, several fatty acids related to oleic acid were shown to alter tmAC activity [43]. For example, oleic acid elevates cAMP in HEK293 cells expressing AC3 but not AC5. Conversely, arachidonic acid selectively attenuates AC1 and AC4. The physiological relevance of AC regulation by fatty acids is not immediately clear, since circulating levels of free fatty acids are low [44]. Despite these exciting discoveries, the field awaits definitive mapping of any cyclase modulator to a TM domain binding site. Furthermore, no conventional primary messengers, such as a hormone or neurotransmitter, have yet been shown to allosterically modulate AC activity via the TM domain.

**Fig. 3:**
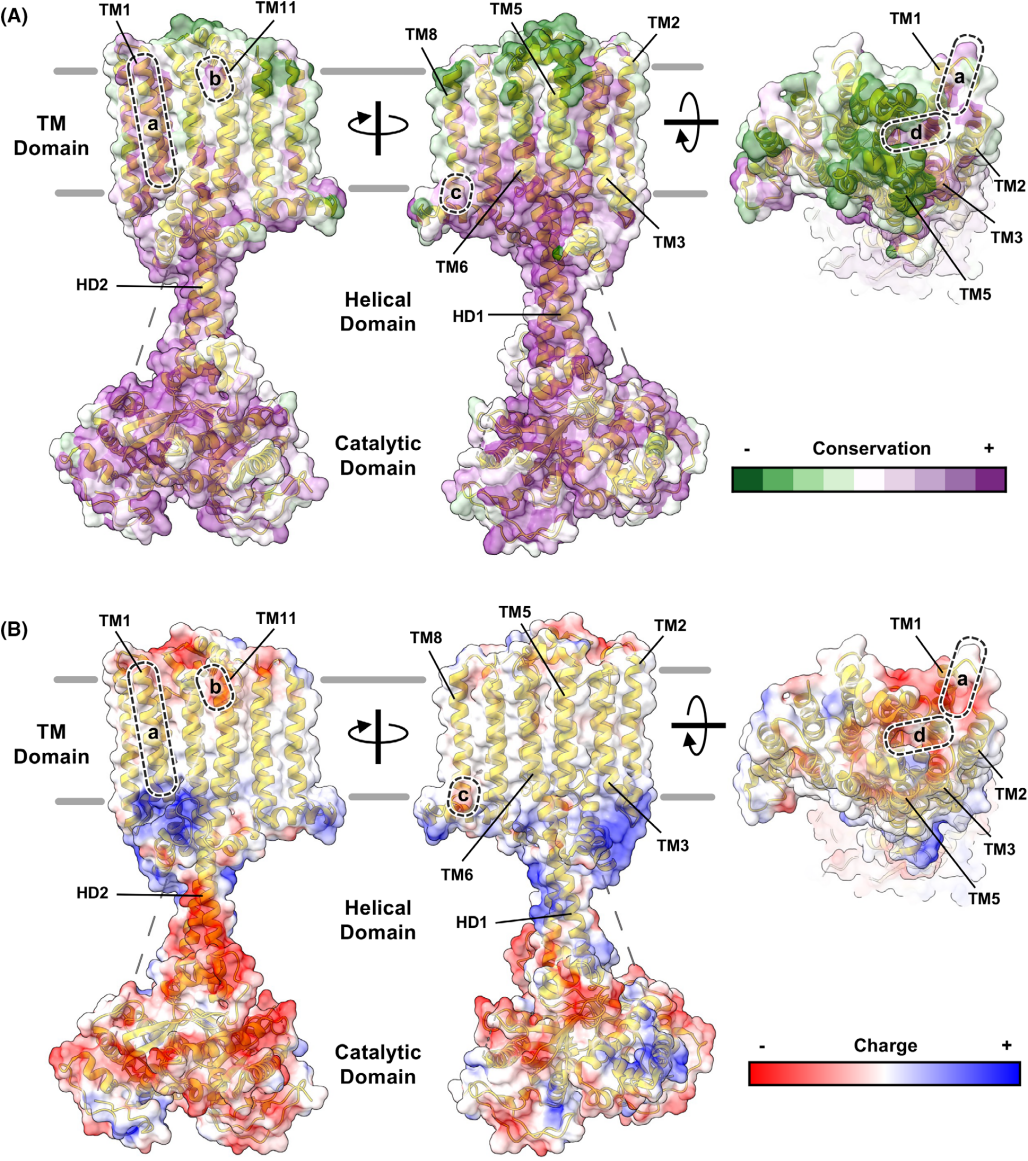
Surface analysis of AC8. Side‐on (left and middle columns) and top‐down (right column) views of the AC8 surface are shown coloured by either sequence conservation (A) or charge (B). Conservation scores for AC8 (PDB 8BUZ) were computed using ConSurf [46], and structural representations were generated using ChimeraX [79]. Homologues with > 50% sequence identity were obtained from the Clean UniProt database, with a total of 482 sequences included in the analysis. The average pairwise distance was 0.36. Four clusters of higher conservation are highlighted: along helix 1 (region a), at the C‐terminus of helix 11 (region b), at the C‐terminus of helix 8 (region c), and in a pocket formed by helix 4 and the loop linking helices 3 and 4 (region d).

Although TM domain sequence conservation across the tmAC family is low [45], more highly conserved pockets and surfaces are evident in alignments across species for specific isoforms [45]. For example, Fig. 3A shows sequence conservation computed using ConSurf [46] overlaid on the structure of AC8 [9]. Intracellular surfaces including the helical and catalytic domains are more highly conserved (purple), whereas the extracellular face of the TM domain is the least conserved (green) with an intermediate level of conservation for the membrane‐embedded surfaces of the TM domain. However, at least four clusters of higher conservation are evident on the AC8 TM domain surface: along helix 1 (region a, Fig. 3A), at the C‐terminus of helix 11 (b), at the C‐terminus of helix 8 (c), and in a pocket formed by helix 4 and a loop between helices 3 and 4 (d). Regions b‐d all include negatively charged side chains (Fig. 3B)—region d is particularly promising as a potential ligand binding site [9]. Molecular dynamics simulations with Rv1625c suggest that a similar negatively charged pocket in this cyclase can bind to positively charged ligands including divalent metal ions [47]. Research on neurosteroid regulation of GABA‐A receptors is instructive [48]. Progress in this field was laboured with extensive use of chimeras, site‐directed mutagenesis and photo‐affinity labelling applied to map binding sites before the advent of cryo‐EM structure determination enabled more conclusive answers. Mapping ligands to AC TM domains should theoretically proceed faster since tmAC research has already decisively entered the cryo‐EM structure era.

## Interactor function

Pre‐assembled signalling complexes are an emerging general feature of signal transduction [49]. cAMP signalling is organised in nanodomains, with close coordination of cyclases, phosphodiesterases and cAMP receptors in complexes often nucleated by AKAPs [50]. However, given that cyclases are the source of all cAMP signals, it might be expected that these enzymes would play a more prominent role in coordinating multiprotein complexes for precise spatiotemporal regulation of cAMP signalling [51]. Given that many important channels and transporters—in addition to all ~ 800 GPCRs [52]—feature bundles of TM helices, there is tremendous scope for interactions between these elements and AC TM helices (Fig. 2D). Multiple tmACs interact with AKAPs including Yotiao [53, 54], AKAP79 [55, 56] and mAKAPβ [57] but these interactions typically involve the cytosolic N‐terminal regulatory regions of the cyclases. There are a few reports of cyclase interactions involving the TM domain. Bioluminescence resonance energy transfer (BRET) and bimolecular fluorescence complementation show that AC5 interacts with both adenosine 2A (A_2A_) and type‐2 dopamine (D_2_R) receptors [58]. Furthermore, disruption experiments with peptides derived from specific TM helices suggest that the interface between AC5 and these receptors depends on the receptor activation state. For example, in the presence of the A_2A_R agonist CGS21680, peptides corresponding to any of the first six TM helices from AC5 disrupt AC5‐A_2A_R interactions, whereas helices 1 and 12 are the most effective disruptors prior to receptor activation [58]. AC9 interacts with POPDC1/2 in the heart through an interaction that involves both TM and cytosolic elements in POPDC [59]. POPDC is thought to bridge AC9 to TREK‐1 channels in this context [59], although the details of the membrane interface have not been established.

Dimerisation is an important feature of tmAC regulation that involves TM domain interactions at least for some isoforms. AC5 undergoes cooperative activation that can be explained by binding to an AC5 dimer [60]. Experiments with truncated cyclases show that the second TM cassette mediates dimer formation for AC8 [31], although a recent homodimeric structure of AC5 suggests that a hairpin loop between the fifth and sixth helices of the C1B domain forms the primary interface rather than the TM helices [8]. Another structure from this same study—a monomeric AC5–Gβγ complex—reveals another interesting potential function for the TM domain. The lipophilic geranylgeranyl group attached to the C‐terminus of Gγ packs against helices 1 and 7 of the TM domain and may thereby support the interaction of the two proteins. It should be noted that although several AC isoforms are localised to lipid rafts, surprisingly the C1 and C2 subdomains are thought to be critical for targeting AC5 and AC8 to these cholesterol‐rich regions [61].

Identifying and studying protein–protein interactions in membranes is challenging [62] not least because complexes may dissociate when cellular membranes are solubilised during conventional techniques for studying protein interactions. There is a growing awareness that GPCRs are regulated by membrane‐embedded accessory proteins [63]. For example, receptor activity‐modifying proteins (RAMPs) are single‐pass proteins that bind family B GPCRs [64]. RAMP2 acts as a negative allosteric modulator of the glucagon receptor with interactions involving membrane‐spanning helices [65]. Similarly, the single‐pass accessory protein MRAP2 biases ghrelin receptor signalling away from β‐arrestin [65]. Research on ion channels including AMPA receptors is also revealing that membrane‐spanning accessory proteins are critical regulators [66]. There are likely to be many unexplored areas in the full landscape of cyclase regulation by membrane accessory proteins. New *in situ* labelling methods are likely to support progress in this endeavour [67].

## Implications for pharmacology

Many potential applications of therapeutic agents targeting ACs have been proposed, for example AC1 is a putative target for treating chronic pain, and AC5 has been put forward as a target for cardioprotection [68]. However, the suitability of ACs as drug targets is limited unless isoform‐selective agents can be developed [68]. A handful of previous screens have been conducted to identify compounds that act on tmACs within crude membranes extracted from HEK293 cells [69, 70, 71]. These screens have identified inhibitors of AC1 [69, 70] and AC2 [71] that compete with ATP for binding to the catalytic domain. A further study focused on fungicide imidazole compounds and identified clotrimazole as a pan‐AC inhibitor without determining its locus of action [38]. Sequence divergence is much higher between tmAC isoforms in the TM than the catalytic subdomains [45], therefore agents that target the TM region are more likely to possess isoform‐specific characteristics. One can speculate on different approaches that could be taken. For example, if drugs can be developed that alter cyclase activity via allosteric sites on the TM domain (Fig. 2C), they might be applied in isolation to selectively elevate or depress cAMP signalling in the desired system. Alternatively, they could be applied in combination with GPCR agonists/antagonists to bias signalling towards either the G protein or arrestin branch [72]. TM domain binders that do not alter cyclase activity might also be useful—they could be fused to GPCR‐directed drugs to increase specificity. Many antibody‐drug conjugate agents are in development [73] including anti‐CD33 antibody conjugated to calicheamicin through acid‐labile linker for the treatment of acute myeloid lymphoma [74]. The recent structure of AC5 highlights how multiple structurally complex polypeptide loops extend outside of the cell membrane for this isoform, which could be amenable to antibody targeting [8]. Finally, interactions mediated by TM domains (Fig. 2D) might be targeted by peptide or small molecule disruptors. This approach has received attention from researchers focused on AKAPs. For example, a peptide has been developed to disrupt interactions between TRPV4 channels and AKAP79 with the aim of treating chronic pain [75], and proof‐of‐concept experiments with a peptide targeting the intracellular AKAP79‐AC5 interface support the viability of this approach [76].

AC TM domains are emerging as important protein interaction hubs, and evidence is accumulating that the domains can serve as receptors. To date, cyclases have been neglected as drug targets in comparison to other components of the cAMP signalling cascade including GPCRs [77] and PDEs [78]. Further efforts to illuminate the AC TM domain function ‘blind spot’ may enable therapeutic strategies targeted at these domains to partly redress this balance in the future.

## Conflict of interest

The authors declare no conflict of interest.

## Author contributions

MGG and RSD wrote the manuscript together; MGG prepared the first two figures; RSD performed the surface analysis of AC8.

## References

[1] Beavo JA & Brunton LL (2002) Cyclic nucleotide research – still expanding after half a century. Nat Rev Mol Cell Biol 3, 710–718. 12209131 10.1038/nrm911

[2] Sutherland EW & Robison GA (1966) The role of cyclic‐3′,5'‐AMP in responses to catecholamines and other hormones. Pharmacol Rev 18, 145–161. 5323765

[3] Lefkowitz RJ (2013) A brief history of G‐protein coupled receptors (Nobel lecture). Angew Chem Int Ed Engl 52, 6366–6378. 23650015 10.1002/anie.201301924

[4] Dessauer CW , Watts VJ , Ostrom RS , Conti M , Dove S & Seifert R (2017) International Union of Basic and Clinical Pharmacology. CI. Structures and small molecule modulators of mammalian adenylyl Cyclases. Pharmacol Rev 69, 93–139. 28255005 10.1124/pr.116.013078 PMC5394921

[5] Schuster D , Khanppnavar B , Kantarci I , Mehta V & Korkhov VM (2024) Structural insights into membrane adenylyl cyclases, initiators of cAMP signaling. Trends Biochem Sci 49, 156–168. 38158273 10.1016/j.tibs.2023.12.002

[6] Tesmer JJ , Sunahara RK , Gilman AG & Sprang SR (1997) Crystal structure of the catalytic domains of adenylyl cyclase in a complex with Gsalpha.GTPgammaS. Science 278, 1907–1916. 9417641 10.1126/science.278.5345.1907

[7] Sunahara RK , Dessauer CW , Whisnant RE , Kleuss C & Gilman AG (1997) Interaction of Gsalpha with the cytosolic domains of mammalian adenylyl cyclase. J Biol Chem 272, 22265–22271. 9268375 10.1074/jbc.272.35.22265

[8] Yen YC , Li Y , Chen CL , Klose T , Watts VJ , Dessauer CW & Tesmer JJG (2024) Structure of adenylyl cyclase 5 in complex with Gbetagamma offers insights into ADCY5‐related dyskinesia. Nat Struct Mol Biol 31, 1189–1197. doi: 10.1038/s41594-024-01263-0 38589608 PMC11329361

[9] Khanppnavar B , Schuster D , Lavriha P , Uliana F , Ozel M , Mehta V , Leitner A , Picotti P & Korkhov VM (2024) Regulatory sites of CaM‐sensitive adenylyl cyclase AC8 revealed by cryo‐EM and structural proteomics. EMBO Rep 25, 1513–1540. 38351373 10.1038/s44319-024-00076-y PMC10933263

[10] Qi C , Sorrentino S , Medalia O & Korkhov VM (2019) The structure of a membrane adenylyl cyclase bound to an activated stimulatory G protein. Science 364, 389–394. 31023924 10.1126/science.aav0778

[11] Krupinski J , Coussen F , Bakalyar HA , Tang WJ , Feinstein PG , Orth K , Slaughter C , Reed RR & Gilman AG (1989) Adenylyl cyclase amino acid sequence: possible channel‐ or transporter‐like structure. Science 244, 1558–1564. 2472670 10.1126/science.2472670

[12] Sen P , Ortiz O , Brivio E , Menegaz D , Sotillos Elliott L , Du Y , Ries C , Chen A , Wurst W , Lopez JP *et al*. (2024) A bipolar disorder‐associated missense variant alters adenylyl cyclase 2 activity and promotes mania‐like behavior. Mol Psychiatry 30, 97–110. 39003412 10.1038/s41380-024-02663-w PMC11649569

[13] Stergiakouli E , Gaillard R , Tavare JM , Balthasar N , Loos RJ , Taal HR , Evans DM , Rivadeneira F , St Pourcain B , Uitterlinden AG *et al*. (2014) Genome‐wide association study of height‐adjusted BMI in childhood identifies functional variant in ADCY3. Obesity (Silver Spring) 22, 2252–2259. 25044758 10.1002/oby.20840 PMC4265207

[14] Zech M , Boesch S , Jochim A , Weber S , Meindl T , Schormair B , Wieland T , Lunetta C , Sansone V , Messner M *et al*. (2017) Clinical exome sequencing in early‐onset generalized dystonia and large‐scale resequencing follow‐up. Mov Disord 32, 549–559. 27666935 10.1002/mds.26808

[15] Waalkens AJE , Vansenne F , van der Hout AH , Zutt R , Mourmans J , Tolosa E , de Koning TJ & Tijssen MAJ (2018) Expanding the ADCY5 phenotype toward spastic paraparesis: a mutation in the M2 domain. Neurol Genet 4, e214. 29473048 10.1212/NXG.0000000000000214 PMC5820596

[16] Yuen RK , Merico D , Cao H , Pellecchia G , Alipanahi B , Thiruvahindrapuram B , Tong X , Sun Y , Cao D , Zhang T *et al*. (2016) Genome‐wide characteristics of de novo mutations in autism. NPJ Genom Med 1, 160271–1602710. 27525107 10.1038/npjgenmed.2016.27 PMC4980121

[17] Magri C , Giacopuzzi E , La Via L , Bonini D , Ravasio V , Elhussiny MEA , Orizio F , Gangemi F , Valsecchi P , Bresciani R *et al*. (2018) A novel homozygous mutation in GAD1 gene described in a schizophrenic patient impairs activity and dimerization of GAD67 enzyme. Sci Rep 8, 15470. 30341396 10.1038/s41598-018-33924-8 PMC6195539

[18] Dessauer CW (2009) Adenylyl cyclase–A‐kinase anchoring protein complexes: the next dimension in cAMP signaling. Mol Pharmacol 76, 935–941. 19684092 10.1124/mol.109.059345 PMC2774998

[19] Qi C , Lavriha P , Mehta V , Khanppnavar B , Mohammed I , Li Y , Lazaratos M , Schaefer JV , Dreier B , Pluckthun A *et al*. (2022) Structural basis of adenylyl cyclase 9 activation. Nat Commun 13, 1045. 35210418 10.1038/s41467-022-28685-y PMC8873477

[20] Alam A & Locher KP (2023) Structure and mechanism of human ABC transporters. Annu Rev Biophys 52, 275–300. 36737602 10.1146/annurev-biophys-111622-091232

[21] Rees DC , Johnson E & Lewinson O (2009) ABC transporters: the power to change. Nat Rev Mol Cell Biol 10, 218–227. 19234479 10.1038/nrm2646 PMC2830722

[22] Miranda ER , Nam EA , Kuspa A & Shaulsky G (2015) The ABC transporter, AbcB3, mediates cAMP export in *D. discoideum* development. Dev Biol 397, 203–211. 25448698 10.1016/j.ydbio.2014.11.006 PMC4277735

[23] Pacini ESA , Satori NA , Jackson EK & Godinho RO (2022) Extracellular cAMP‐adenosine pathway signaling: a potential therapeutic target in chronic inflammatory airway diseases. Front Immunol 13, 866097. 35479074 10.3389/fimmu.2022.866097 PMC9038211

[24] Pourmal S , Green E , Bajaj R , Chemmama IE , Knudsen GM , Gupta M , Sali A , Cheng Y , Craik CS , Kroetz DL *et al*. (2024) Structural basis of prostaglandin efflux by MRP4. Nat Struct Mol Biol 31, 621–632. 38216659 10.1038/s41594-023-01176-4 PMC11145372

[25] Schultz JE , Klumpp S , Benz R , Schurhoff‐Goeters WJ & Schmid A (1992) Regulation of adenylyl cyclase from *Paramecium* by an intrinsic potassium conductance. Science 255, 600–603. 1371017 10.1126/science.1371017

[26] Weber JH , Vishnyakov A , Hambach K , Schultz A , Schultz JE & Linder JU (2004) Adenylyl cyclases from *Plasmodium*, *Paramecium* and *Tetrahymena* are novel ion channel/enzyme fusion proteins. Cell Signal 16, 115–125. 14607282 10.1016/s0898-6568(03)00129-3

[27] Reddy R , Smith D , Wayman G , Wu Z , Villacres EC & Storm DR (1995) Voltage‐sensitive adenylyl cyclase activity in cultured neurons. A calcium‐independent phenomenon. J Biol Chem 270, 14340–14346. 7782293 10.1074/jbc.270.24.14340

[28] Cooper DM , Schell MJ , Thorn P & Irvine RF (1998) Regulation of adenylyl cyclase by membrane potential. J Biol Chem 273, 27703–27707. 9765307 10.1074/jbc.273.42.27703

[29] Catterall WA , Lenaeus MJ & Gamal El‐Din TM (2020) Structure and pharmacology of voltage‐gated sodium and calcium channels. Annu Rev Pharmacol Toxicol 60, 133–154. 31537174 10.1146/annurev-pharmtox-010818-021757

[30] Boutonnet M , Bunemann M & Perroy J (2024) The voltage sensitivity of G‐protein coupled receptors: unraveling molecular mechanisms and physiological implications. Pharmacol Ther 264, 108741. 39489434 10.1016/j.pharmthera.2024.108741

[31] Gu C , Sorkin A & Cooper DM (2001) Persistent interactions between the two transmembrane clusters dictate the targeting and functional assembly of adenylyl cyclase. Curr Biol 11, 185–190. 11231154 10.1016/s0960-9822(01)00044-6

[32] Seebacher T , Linder JU & Schultz JE (2001) An isoform‐specific interaction of the membrane anchors affects mammalian adenylyl cyclase type V activity. Eur J Biochem 268, 105–110. 11121109 10.1046/j.1432-1327.2001.01850.x

[33] Vercellino I , Rezabkova L , Olieric V , Polyhach Y , Weinert T , Kammerer RA , Jeschke G & Korkhov VM (2017) Role of the nucleotidyl cyclase helical domain in catalytically active dimer formation. Proc Natl Acad Sci USA 114, E9821–E9828. 29087332 10.1073/pnas.1712621114 PMC5699072

[34] Parent CA & Devreotes PN (1996) Constitutively active adenylyl cyclase mutant requires neither G proteins nor cytosolic regulators. J Biol Chem 271, 18333–18336. 8702473 10.1074/jbc.271.31.18333

[35] Kang Y , Liu R , Wu JX & Chen L (2019) Structural insights into the mechanism of human soluble guanylate cyclase. Nature 574, 206–210. 31514202 10.1038/s41586-019-1584-6

[36] Beltz S , Bassler J & Schultz JE (2016) Regulation by the quorum sensor from vibrio indicates a receptor function for the membrane anchors of adenylate cyclases. elife 5, e13098. 26920221 10.7554/eLife.13098 PMC4821796

[37] Seth A , Finkbeiner M , Grischin J & Schultz JE (2020) Gsalpha stimulation of mammalian adenylate cyclases regulated by their hexahelical membrane anchors. Cell Signal 68, 109538. 31931092 10.1016/j.cellsig.2020.109538

[38] Simpson J , Palvolgyi A & Antoni FA (2019) Direct stimulation of adenylyl cyclase 9 by the fungicide imidazole miconazole. Naunyn Schmiedeberg's Arch Pharmacol 392, 497–504. 30607468 10.1007/s00210-018-01610-1

[39] Haunso A , Simpson J & Antoni FA (2003) Small ligands modulating the activity of mammalian adenylyl cyclases: a novel mode of inhibition by calmidazolium. Mol Pharmacol 63, 624–631. 12606770 10.1124/mol.63.3.624

[40] Duncan AL , Song W & Sansom MSP (2020) Lipid‐dependent regulation of ion channels and G protein‐coupled receptors: insights from structures and simulations. Annu Rev Pharmacol Toxicol 60, 31–50. 31506010 10.1146/annurev-pharmtox-010919-023411

[41] Seth A , Landau M , Shevchenko A , Traikov S , Schultz A , Elsabbagh S & Schultz JE (2022) Distinct glycerophospholipids potentiate Gsalpha‐activated adenylyl cyclase activity. Cell Signal 97, 110396. 35787445 10.1016/j.cellsig.2022.110396

[42] Elsabbagh S , Landau M , Gross H , Schultz A & Schultz JE (2023) Heme b inhibits class III adenylyl cyclases. Cell Signal 103, 110568. 36565898 10.1016/j.cellsig.2022.110568

[43] Landau M , Elsabbagh S , Gross H , Fuchs ACD , Schultz ACF & Schultz JE (2024) The membrane domains of mammalian adenylyl cyclases are lipid receptors. elife 13, RP101483. 39611663 10.7554/eLife.101483 PMC11606603

[44] Huber AH & Kleinfeld AM (2017) Unbound free fatty acid profiles in human plasma and the unexpected absence of unbound palmitoleate. J Lipid Res 58, 578–585. 28082409 10.1194/jlr.M074260 PMC5335587

[45] Schultz JE (2022) The evolutionary conservation of eukaryotic membrane‐bound adenylyl cyclase isoforms. Front Pharmacol 13, 1009797. 36238545 10.3389/fphar.2022.1009797 PMC9552081

[46] Yariv B , Yariv E , Kessel A , Masrati G , Chorin AB , Martz E , Mayrose I , Pupko T & Ben‐Tal N (2023) Using evolutionary data to make sense of macromolecules with a “face‐lifted” ConSurf. Protein Sci 32, e4582. 36718848 10.1002/pro.4582 PMC9942591

[47] Mehta V , Khanppnavar B , Schuster D , Kantarci I , Vercellino I , Kosturanova A , Iype T , Stefanic S , Picotti P & Korkhov VM (2022) Structure of *Mycobacterium tuberculosis* Cya, an evolutionary ancestor of the mammalian membrane adenylyl cyclases. elife 11, e77032. 35980026 10.7554/eLife.77032 PMC9433096

[48] Mortensen M , Bright DP , Fagotti J , Dorovykh V , Cerna B & Smart TG (2024) Forty years searching for neurosteroid binding sites on GABA(a) receptors. Neuroscience doi: 10.1016/j.neuroscience.2024.06.002 38852898

[49] Ferre S , Ciruela F , Dessauer CW , Gonzalez‐Maeso J , Hebert TE , Jockers R , Logothetis DE & Pardo L (2022) G protein‐coupled receptor‐effector macromolecular membrane assemblies (GEMMAs). Pharmacol Ther 231, 107977. 34480967 10.1016/j.pharmthera.2021.107977 PMC9375844

[50] Bock A , Irannejad R & Scott JD (2024) cAMP signaling: a remarkably regional affair. Trends Biochem Sci 49, 305–317. 38310024 10.1016/j.tibs.2024.01.004 PMC11175624

[51] Zaccolo M , Zerio A & Lobo MJ (2021) Subcellular organization of the cAMP signaling pathway. Pharmacol Rev 73, 278–309. 33334857 10.1124/pharmrev.120.000086 PMC7770493

[52] Weis WI & Kobilka BK (2018) The molecular basis of G protein‐coupled receptor activation. Annu Rev Biochem 87, 897–919. 29925258 10.1146/annurev-biochem-060614-033910 PMC6535337

[53] Li Y , Chen L , Kass RS & Dessauer CW (2012) The A‐kinase anchoring protein Yotiao facilitates complex formation between adenylyl cyclase type 9 and the IKs potassium channel in heart. J Biol Chem 287, 29815–29824. 22778270 10.1074/jbc.M112.380568 PMC3436180

[54] Piggott LA , Bauman AL , Scott JD & Dessauer CW (2008) The A‐kinase anchoring protein Yotiao binds and regulates adenylyl cyclase in brain. Proc Natl Acad Sci USA 105, 13835–13840. 18772391 10.1073/pnas.0712100105 PMC2544540

[55] Bauman AL , Soughayer J , Nguyen BT , Willoughby D , Carnegie GK , Wong W , Hoshi N , Langeberg LK , Cooper DM , Dessauer CW *et al*. (2006) Dynamic regulation of cAMP synthesis through anchored PKA‐adenylyl cyclase V/VI complexes. Mol Cell 23, 925–931. 16973443 10.1016/j.molcel.2006.07.025 PMC3941446

[56] Efendiev R , Samelson BK , Nguyen BT , Phatarpekar PV , Baameur F , Scott JD & Dessauer CW (2010) AKAP79 interacts with multiple adenylyl cyclase (AC) isoforms and scaffolds AC5 and ‐6 to alpha‐amino‐3‐hydroxyl‐5‐methyl‐4‐isoxazole‐propionate (AMPA) receptors. J Biol Chem 285, 14450–14458. 20231277 10.1074/jbc.M110.109769 PMC2863235

[57] Kapiloff MS , Piggott LA , Sadana R , Li J , Heredia LA , Henson E , Efendiev R & Dessauer CW (2009) An adenylyl cyclase‐mAKAPbeta signaling complex regulates cAMP levels in cardiac myocytes. J Biol Chem 284, 23540–23546. 19574217 10.1074/jbc.M109.030072 PMC2749128

[58] Navarro G , Cordomi A , Casado‐Anguera V , Moreno E , Cai NS , Cortes A , Canela EI , Dessauer CW , Casado V , Pardo L *et al*. (2018) Evidence for functional pre‐coupled complexes of receptor heteromers and adenylyl cyclase. Nat Commun 9, 1242. 29593213 10.1038/s41467-018-03522-3 PMC5871782

[59] Baldwin TA , Li Y , Marsden AN , Rinne S , Garza‐Carbajal A , Schindler RFR , Zhang M , Garcia MA , Venna VR , Decher N *et al*. (2022) POPDC1 scaffolds a complex of adenylyl cyclase 9 and the potassium channel TREK‐1 in heart. EMBO Rep 23, e55208. 36254885 10.15252/embr.202255208 PMC9724675

[60] Chen‐Goodspeed M , Lukan AN & Dessauer CW (2005) Modeling of Galpha(s) and Galpha(i) regulation of human type V and VI adenylyl cyclase. J Biol Chem 280, 1808–1816. 15545274 10.1074/jbc.M409172200

[61] Crossthwaite AJ , Seebacher T , Masada N , Ciruela A , Dufraux K , Schultz JE & Cooper DM (2005) The cytosolic domains of Ca^2+^−sensitive adenylyl cyclases dictate their targeting to plasma membrane lipid rafts. J Biol Chem 280, 6380–6391. 15574428 10.1074/jbc.M411987200

[62] Duart G , Grau B , Mingarro I & Martinez‐Gil L (2021) Methodological approaches for the analysis of transmembrane domain interactions: a systematic review. Biochim Biophys Acta Biomembr 1863, 183712. 34331948 10.1016/j.bbamem.2021.183712

[63] Zhang M , Chen T , Lu X , Lan X , Chen Z & Lu S (2024) G protein‐coupled receptors (GPCRs): advances in structures, mechanisms, and drug discovery. Signal Transduct Target Ther 9, 88. 38594257 10.1038/s41392-024-01803-6 PMC11004190

[64] Wang M , Lyu J & Zhang C (2024) Single transmembrane GPCR modulating proteins: neither single nor simple. Protein Cell 15, 395–402. 37314044 10.1093/procel/pwad035 PMC11131010

[65] Rouault AAJ , Rosselli‐Murai LK , Hernandez CC , Gimenez LE , Tall GG & Sebag JA (2020) The GPCR accessory protein MRAP2 regulates both biased signaling and constitutive activity of the ghrelin receptor GHSR1a. Sci Signal 13, eaax4569. 31911434 10.1126/scisignal.aax4569 PMC7291826

[66] Greger IH , Watson JF & Cull‐Candy SG (2017) Structural and functional architecture of AMPA‐type glutamate receptors and their auxiliary proteins. Neuron 94, 713–730. 28521126 10.1016/j.neuron.2017.04.009

[67] Zafra F & Piniella D (2022) Proximity labeling methods for proteomic analysis of membrane proteins. J Proteome 264, 104620. 10.1016/j.jprot.2022.104620 35598870

[68] Ostrom KF , LaVigne JE , Brust TF , Seifert R , Dessauer CW , Watts VJ & Ostrom RS (2022) Physiological roles of mammalian transmembrane adenylyl cyclase isoforms. Physiol Rev 102, 815–857. 34698552 10.1152/physrev.00013.2021 PMC8759965

[69] Giacoletti G , Price T , Hoelz LVB , Shremo Msdi A , Cossin S , Vazquez‐Falto K , Amorim Fernandes TV , Santos de Pontes V , Wang H , Boechat N *et al*. (2022) A selective adenylyl cyclase 1 inhibitor relieves pain without causing tolerance. Front Pharmacol 13, 935588. 35899113 10.3389/fphar.2022.935588 PMC9310748

[70] Brust TF , Alongkronrusmee D , Soto‐Velasquez M , Baldwin TA , Ye Z , Dai M , Dessauer CW , van Rijn RM & Watts VJ (2017) Identification of a selective small‐molecule inhibitor of type 1 adenylyl cyclase activity with analgesic properties. Sci Signal 10. 10, eaah5381. 28223412 10.1126/scisignal.aah5381 PMC5734633

[71] Conley JM , Brand CS , Bogard AS , Pratt EP , Xu R , Hockerman GH , Ostrom RS , Dessauer CW & Watts VJ (2013) Development of a high‐throughput screening paradigm for the discovery of small‐molecule modulators of adenylyl cyclase: identification of an adenylyl cyclase 2 inhibitor. J Pharmacol Exp Ther 347, 276–287. 24008337 10.1124/jpet.113.207449 PMC3807067

[72] Che T , Dwivedi‐Agnihotri H , Shukla AK & Roth BL (2021) Biased ligands at opioid receptors: current status and future directions. Sci Signal 14, eaav0320. 33824179 10.1126/scisignal.aav0320 PMC7611221

[73] Zhao Z , Ukidve A , Kim J & Mitragotri S (2020) Targeting strategies for tissue‐specific drug delivery. Cell 181, 151–167. 32243788 10.1016/j.cell.2020.02.001

[74] Norsworthy KJ , Ko CW , Lee JE , Liu J , John CS , Przepiorka D , Farrell AT & Pazdur R (2018) FDA approval summary: Mylotarg for treatment of patients with relapsed or refractory CD33‐positive acute myeloid leukemia. Oncologist 23, 1103–1108. 29650683 10.1634/theoncologist.2017-0604 PMC6192608

[75] Mack K & Fischer MJM (2017) Disrupting sensitization of TRPV4. Neuroscience 352, 1–8. 28372987 10.1016/j.neuroscience.2017.03.037

[76] Bavencoffe A , Li Y , Wu Z , Yang Q , Herrera J , Kennedy EJ , Walters ET & Dessauer CW (2016) Persistent electrical activity in primary nociceptors after spinal cord injury is maintained by Scaffolded adenylyl cyclase and protein kinase a and is associated with altered adenylyl cyclase regulation. J Neurosci 36, 1660–1668. 26843647 10.1523/JNEUROSCI.0895-15.2016 PMC4737775

[77] Hauser AS , Attwood MM , Rask‐Andersen M , Schioth HB & Gloriam DE (2017) Trends in GPCR drug discovery: new agents, targets and indications. Nat Rev Drug Discov 16, 829–842. 29075003 10.1038/nrd.2017.178 PMC6882681

[78] Baillie GS , Tejeda GS & Kelly MP (2019) Therapeutic targeting of 3′,5′‐cyclic nucleotide phosphodiesterases: inhibition and beyond. Nat Rev Drug Discov 18, 770–796. 31388135 10.1038/s41573-019-0033-4 PMC6773486

[79] Meng EC , Goddard TD , Pettersen EF , Couch GS , Pearson ZJ , Morris JH & Ferrin TE (2023) UCSF ChimeraX: tools for structure building and analysis. Protein Sci 32, e4792. 37774136 10.1002/pro.4792 PMC10588335

